# Effect of Statins on Survival Following Stroke in Patients With Cancer

**DOI:** 10.3389/fneur.2018.00205

**Published:** 2018-04-24

**Authors:** Ye Sel Kim, Moo-Seok Park, Jun-Hwa Lee, Jong-Won Chung, Mi Ji Lee, Chi Kyung Kim, Jin-Man Jung, Kyungmi Oh, Oh Young Bang, Geong-Moon Kim, Ji-Mi Choi, Juneyoung Lee, Chin Sang Chung, Kwang Ho Lee, Woo-Keun Seo

**Affiliations:** ^1^Department of Neurology, Samsung Medical Center, School of Medicine, Sungkyunkwan University, Seoul, South Korea; ^2^Department of Neurology, Korea University Kuro Hospital, College of Medicine, Korea University, Seoul, South Korea; ^3^Department of Neurology, Korea University Ansan Hospital, College of Medicine, Korea University, Seoul, South Korea; ^4^Department of Biostatics, Korea University College of Medicine, Seoul, South Korea; ^5^Department of Digital Health, The Samsung Advanced Institute for Health Sciences and Technology (SAIHST), Sungkyunkwan University, Seoul, South Korea

**Keywords:** outcome, stroke, cancer, statin, mortality

## Abstract

The objective of this study was to investigate the potential benefits of statin therapy initiation in acute stroke in patients with active cancer. This study was conducted in two parts. First, data from patients who are presented with stroke and active cancer were obtained from prospectively collected multicenter hospital-based stroke registries. Patients were classified into statin user and non-user groups; the statin group was further divided into low-potency and high-potency statin subgroups. The primary outcome was time to mortality. Second, we obtained data from the Korean National Health Information Service-National Sample Cohort (NHIS-NSC) database for external validation and analyzed the effect of statins on mortality, taking compliance into consideration. For the stroke registry cohort, statin use was independently associated with reduced mortality in a multivariable model [hazard ratio (HR) = 0.675, 95% confidence interval (CI) = 0.457–0.996]. There was no interaction between statin use and cancer characteristics, vascular risk factors, or laboratory findings. A dose-dependent relationship between statin use and survival was also demonstrated. Analysis of the NHIS-NSC database found a similar association between statin therapy and reduced mortality (adjusted HR = 0.64, 95% CI = 0.45–0.90) and this effect persisted even after controlling for the adherence of statin use (HR = 0.60, 95% CI = 0.41–0.89). Statin therapy could be associated with reduced mortality in patients with acute stroke and active cancer.

## Introduction

Statins, or 3-hydroxy-3-methylglutaryl-coenzyme A reductase inhibitors, reduce the primary and secondary risk of stroke and stroke mortality (1). The beneficial effect of statins on stroke prevention is produced by lowering low-density lipoprotein cholesterol (LDL-C) levels, and thereby slowing the progression of atherosclerosis and plaque formation (1, 2). Besides its ability to alter lipid metabolism, retrospective studies have found that statin use improved the outcome of acute ischemic strokes, and this acute effect of statin is thought to be a result of its neuroprotective pleiotropic properties, such as vasodilatory, anti-thrombotic, and anti-inflammatory actions (3).

The pleiotropic effect of statins has also been extensively studied in non-cardiovascular diseases. Interestingly, both *in vitro* and *in vivo* studies have reported the anticancer property of statins (4). In contrast to concerns with the potential carcinogenicity of statins from early animal and epidemiologic studies (5, 6), researchers have found that statin therapy was related to reduce cancer occurrence and better survival of patients with cancer (4). A large meta-analysis from Women’s Health Initiatives demonstrated the beneficial effect of statins on cancer, finding that regular use of statin or other lipid-lowering medications was associated with decreased cancer death, regardless of the type, duration, or potency of statin medication used (7).

Whether due to its neuroprotective effect or anti-neoplastic potential, statins may have a positive impact on patients with both acute stroke and cancer. In this study, we investigated the effect of post-stroke statin therapy on the survival of patients with cancer and acute stroke, using multicenter stroke registries and nation-wide health insurance service data.

## Materials and Methods

### Standard Protocol Approval, Registrations, and Patient Consent

This study was conducted in two parts. First, a retrospective analysis was performed to evaluate the effect of statins on survival among patients with acute stroke and cancer using prospectively collected stroke registries. The second part consisted of the external validation of the findings from the first part, using information obtained from the Korean National Health Insurance Service-National Sample Cohort (NHIS-NSC) database (8).

The study protocol was approved and supervised by the Institutional Review Boards (IRB) of Samsung Medical Center, Korea University Guro Hospital, and Korea University Ansan Hospital. Stroke registries were collected after obtaining informed consent from patients themselves or their family members during admission. Since this was a retrospective observational study using an anonymized database, the IRB granted a waiver to conduct this study without written informed consent.

### Study Populations for Hospital Registry-Based Observational Study

The first part of this study was a multicenter stroke registry-based observational study. Data were obtained from a dataset that comprised two different prospectively collected acute stroke registries from three centers (Samsung Medical Center Stroke Registry and Korea University Stroke Registry-Guro and Ansan arms) for the period between May 2006 and June 2015. The details of these registries can be found elsewhere (9, 10). Both registries included demographic, clinical, and laboratory data from acute stroke patients that were collected within 7 days of the onset of stroke. Both registries employed the same criteria for acute stroke, i.e., either a transient or permanent neurological symptom accompanied by positive relevant lesions on brain imaging.

From the multicenter stroke registry database, we selected patients with active cancer defined as in previous study: a diagnosis of cancer in the 6 months prior to enrollment, any treatment for cancer within the previous 6 months, or recurrent or metastatic cancer (11). The patients were then classified according to severity (with metastasis versus without metastasis) and histological types of cancer (adenocarcinoma versus non-adenocarcinoma). Due to heterogeneity, cancer location was removed from the main analysis and included as a variable for subgroup analysis.

Since the primary purpose of this study was to elucidate the effect of statins, we divided patients into statin user and non-user groups. Patients in the statin user group were those prescribed statins at discharge, who were then further divided into high-potency and low-potency statin users. The high-potency statin group was defined by an expected LDL-C reduction greater than 50% of baseline (atorvastatin 40 mg, atorvastatin 80 mg, rosuvastatin 10 mg, and rosuvastatin 20 mg) (12). Prior statin use was included as a variable in statistical analyses. Both former statin users and non-users were included in this study.

### Clinical and Laboratory Assessment

Stroke severity was assessed using the National Institutes of Health Stroke Scale (NIHSS) scores and modified Rankin score (mRS) at 7 days (or at discharge). According to the Stop Stroke Study Trial of Org 10172 classification (13), patients with evident stroke etiologies, such as large artery atherosclerosis, small artery occlusion, and cardioaortic embolism, were classified as the “conventional stroke group.” The subjects without evident conventional stroke etiologies were classified as the “cryptogenic stroke group.” Cancer was not considered an “other determined etiology” in this study.

Vascular risk factors assessed in this study included hypertension, diabetes mellitus, and atrial fibrillation. Laboratory data from the day of admission or the next day were obtained using blood samples collected after at least 8 h of fasting and included hemoglobin, white blood cell count, platelet count, total cholesterol, and high sensitivity C-reactive protein (hs-CRP). The proportion of subjects with previous statin therapy and anticoagulation therapy at discharge was calculated.

### Outcome Assessment

The primary outcome in this study was time to mortality from any cause. To measure mortality as an acute stroke outcome, we only considered survival data for 3 years. Those lost to follow-up before 3 years were treated as censored data. Data regarding patient outcomes were obtained *via* regular outpatient visits by patients or, if patient was unable to visit the outpatient clinic, telephone interviews conducted by a trained interviewer.

### Statistical Analysis

All statistical analyses were performed using IBM SPSS Statistics for Windows, version 19 (IBM Corp., Armonk, NY, USA), and SAS version 9.2 (SAS Institute, Inc., Cary, NC, USA). Continuous variables are presented as mean ± SD. Categorical variables are presented as a number with percentages. Between-group comparisons were performed using the chi-square, Student’s *t*-test, analysis of variance, or Kruskal–Wallis tests according to the distribution of the variables. To investigate the association between statin therapy and mortality, analyses using univariate and multivariate Cox’s proportional hazard model were employed. Variables that were significantly associated with survival by univariate methods were used in a multivariate regression model using a backward stepwise method. Furthermore, a Kaplan–Meier’s survival curve analysis was used for demonstrating the effect of statin therapy on mortality at year one, two, and three.

In addition, subgroup analyses using an interaction term were performed to test whether the effect of statins was consistent for different subgroups. Patients were dichotomized according to age (cutoff value, 65 years), sex (male or female), location of primary cancer (lung or other locations), mRS at discharge (cutoff value, 2), hemoglobin level (cutoff value, 12 mg/mL), hs-CRP (cutoff value, 2 mg/mL), total cholesterol (cutoff value, 165 mg/dL), and the presence of hypertension, diabetes mellitus, atrial fibrillation, smoking, adenocarcinoma, metastasis, previous statin use, and anticoagulation at discharge. At this time, we added two additional laboratory values, LDL-C and D-dimer, which were excluded from the main analyses because 26 and 60 patients did not have these values recorded, respectively. The cutoff value for low and high D-dimer levels was set at 5 µg/mL for subgroup analysis.

### NHIS-NSC Database

In the second part of this study, national sample cohort information was obtained from the Korean NHIS-NSC database. The NHIS system is a public health insurance system that covers the entire Korean population. The NHIS provided this information as part of the NHIS-NSC open database for health-related research (8). Since it is anonymous information, a waiver of individual consent was granted by the IRB.

**Figure 1:**
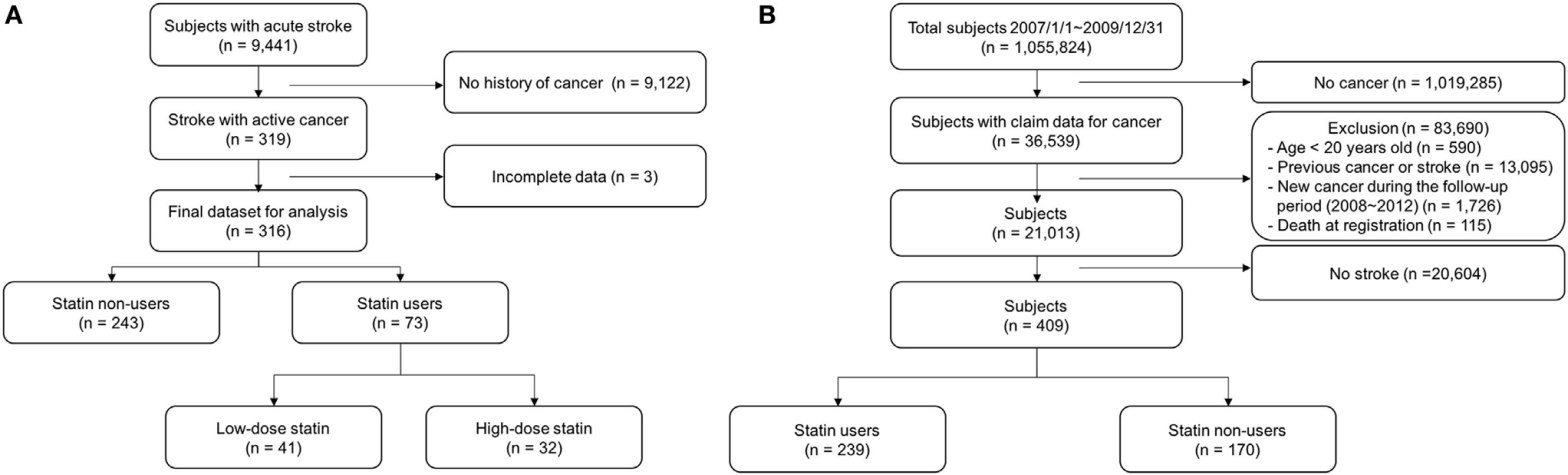
Flow chart for patient selection from the stroke registry cohort **(A)** and National Health Insurance-National Sample Cohort database **(B)**.

A schematic of the study design for the NHIS-NSC database is illustrated in Figure 1B. From the yearly sampled database (*n* = 1,055,824; 2007/1/1–2009/12/31), we obtained information concerning the baseline characteristics, medication, and survival of the target subjects. Subjects who were younger than 20 years or had any history of cancer or ischemic stroke between 2002 and 2006 were excluded from the study. “Stroke with cancer” was defined as an ischemic stroke that occurred within the year prior (to include patients with overt cancer at stroke onset) to up to 5 years after the diagnosis of cancer. Therefore, we selected patients with the following two criteria: (1) those who received a diagnosis of cancer (International Codes of Disease 10th Edition or ICD-10 code C00-C97) and NHIS code V193, which is applied to pathologically confirmed cancer patients in NHIS of Korea during the 3-year period (from January, 2007 to December, 2009), and (2) those who received a diagnosis of ischemic stroke from 1 year prior to cancer diagnosis to up to 5 years after the cancer diagnosis. Since acute ischemic stroke could not be discriminated from chronic or silent ischemic stroke using brain imaging data with ICD-10 codes, we used an operational definition of ischemic stroke to elaborate the diagnosis of acute ischemic stroke as follows: either (1) the presence of at least 3 admission days, at least 1 brain image, and a history of receiving either anti-thrombotic therapies within 1 month, or (2) receiving intravenous tissue plasminogen-activator infusion or intra-arterial recanalization therapy.

Cox’s regression analyses were applied to test the effect of statins on survival. We particularly tested statin adherence to verify the effect of statins on the outcome of acute stroke patients with active cancer. Adherence was calculated by dividing the total number of statin prescription days by the time from the onset of stroke to death or end of the study (%).

## Results

### Stroke Registry Cohort

For the stroke registry cohort, out of a total of 9,441 patients who were registered during the study period, 316 patients (male 59.3%, average age 66.2 ± 10.6 years old) with active cancer were selected for analysis (Figure 1A). During the 3-year follow up period, a total of 228 (72.2%) patients died, 190 (78.2%) and 38 (52.1%) patients in the statin non-user and user groups, respectively. The mean duration of follow-up for the 316 patients was 10.8 months (median = 4.4 months, interquartile range = 1.5–16.9 months). Forty-four (13.9%) patients were lost to follow-up and considered as censored at the end of the follow-up date. The cumulative mortality rate was 64, 74, and 78% at the end of the first, second, and third year, respectively.

Baseline characteristics in statin users and non-users are presented in Table 1. Patients who were prescribed statins (*n* = 73, 23.1%) were older, had a higher proportion of hypertension and pre-stroke statin use, and higher body mass index, hemoglobin level, platelet count, and total cholesterol than statin non-users. In contrast, the proportion of patients with anticoagulation at discharge or metastasis was higher in statin non-users.

**Table 1:** Baseline characteristics of the subjects categorized according to statin therapy.

	Statin non-user (*n* = 243)	Statin user (*n* = 73)	Total (*n* = 316)	*p*-Values
Age	65.41 ± 10.48	68.6 ± 10.84	66.19 ± 10.64	0.018
Sex, male	144 (59.3)	46 (63.0)	190 (60.2)	0.589
Body mass index, kg/m^2^	21.89 ± 3.72	23.58 ± 2.97	22.28 ± 0.20	<0.001
Hypertension	97 (39.9)	39 (53.5)	136 (43.0)	0.044
Diabetes mellitus	57 (23.5)	22 (30.1)	79 (25.0)	0.281
Atrial fibrillation	24 (9.9)	7 (9.6)	31 (9.8)	1.000
Smoking	49 (29.2)	17 (23.3)	66 (20.9)	0.623
Hemoglobin, g/dl	11.42 ± 2.49	12.16 ± 2.14	11.59 ± 2.43	0.022
White blood cell count, 10^3^/μL	9.26 ± 7.02	8.53 ± 3.23	9.09 ± 6.35	0.384
Platelet count, 10^3^/μL	182.0 ± 105.7	235.8 ± 104.6	194.4 ± 107.7	<0.001
C-reactive protein, mg/dl	7.48 ± 23.78	6.37 ± 14.34	7.22 ± 21.93	0.705
Total cholesterol, mg/dl	159.77 ± 45.06	176.47 ± 60.32	163.63 ± 49.41	0.011
NIHSS at admission	6.79 ± 6.67	5.65 ± 6.20	6.53 ± 6.57	0.196
Modified Rankin score 0–2 at 7 days	144 (59.3)	50 (68.5)	194 (61.4)	0.155
Anticoagulation at discharge	173 (71.2)	34 (46.6)	207 (65.5)	<0.001
Pre-stroke statin use	4 (1.6)	25 (34.2)	29 (9.2)	<0.001
Adenocarcinoma	146 (60.1)	35 (47.9)	181 (57.3)	0.079
Metastasis	153 (63.0)	34 (46.6)	187 (59.2)	0.015
Stroke mechanism				<0.001
Conventional	66 (27.2)	45 (61.6)	111 (35.1)	
Cryptogenic	177 (72.8)	28 (38.4)	205 (64.9)	

*NIHSS, National Institutes of Health Stroke Scale*.

*Values are mean ± SD or number (%)*.

**Figure 2:**
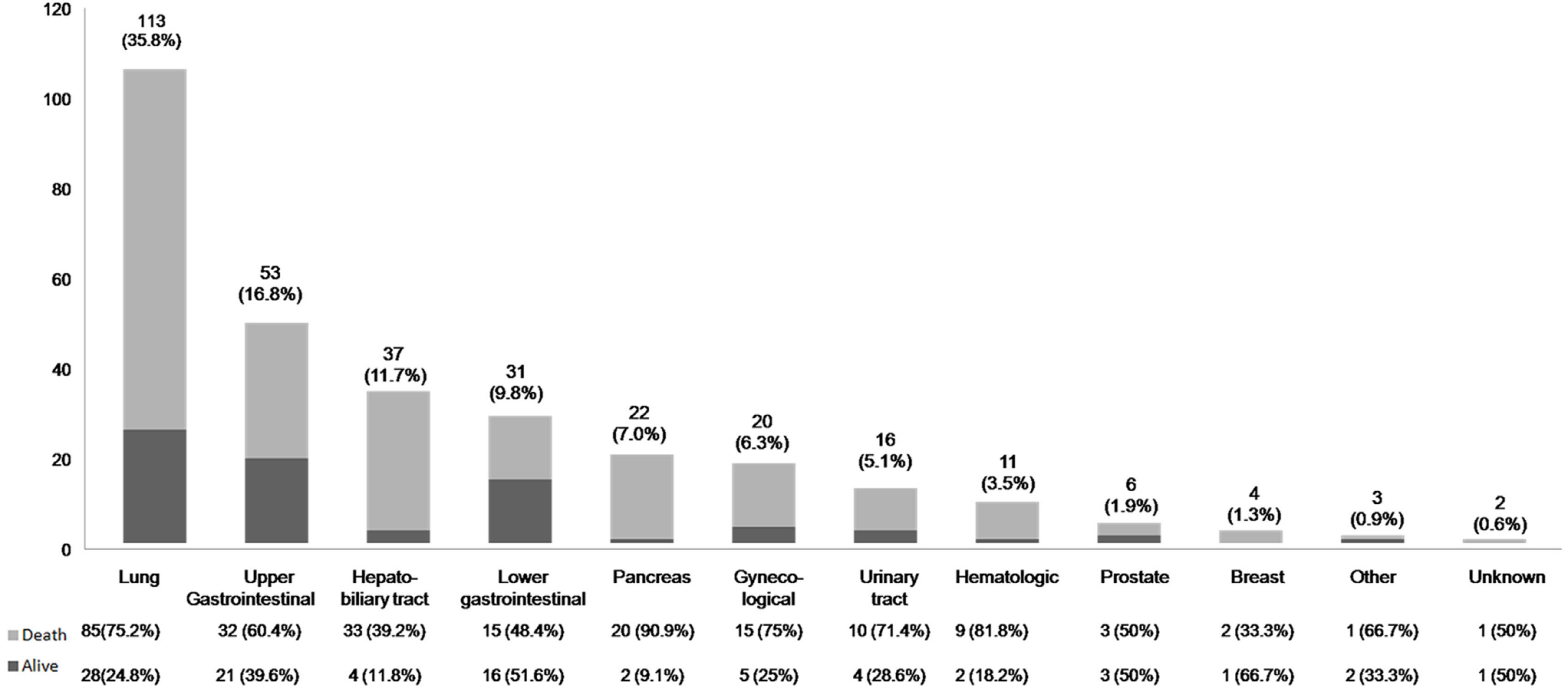
Distribution of location of primary cancers and the survival outcome.

In terms of cancer location, lung cancers were the most common type of cancer, followed by upper gastrointestinal tract and hepatobiliary cancers. The survival outcome by cancer location is outlined in Figure 2.

**Figure 3:**
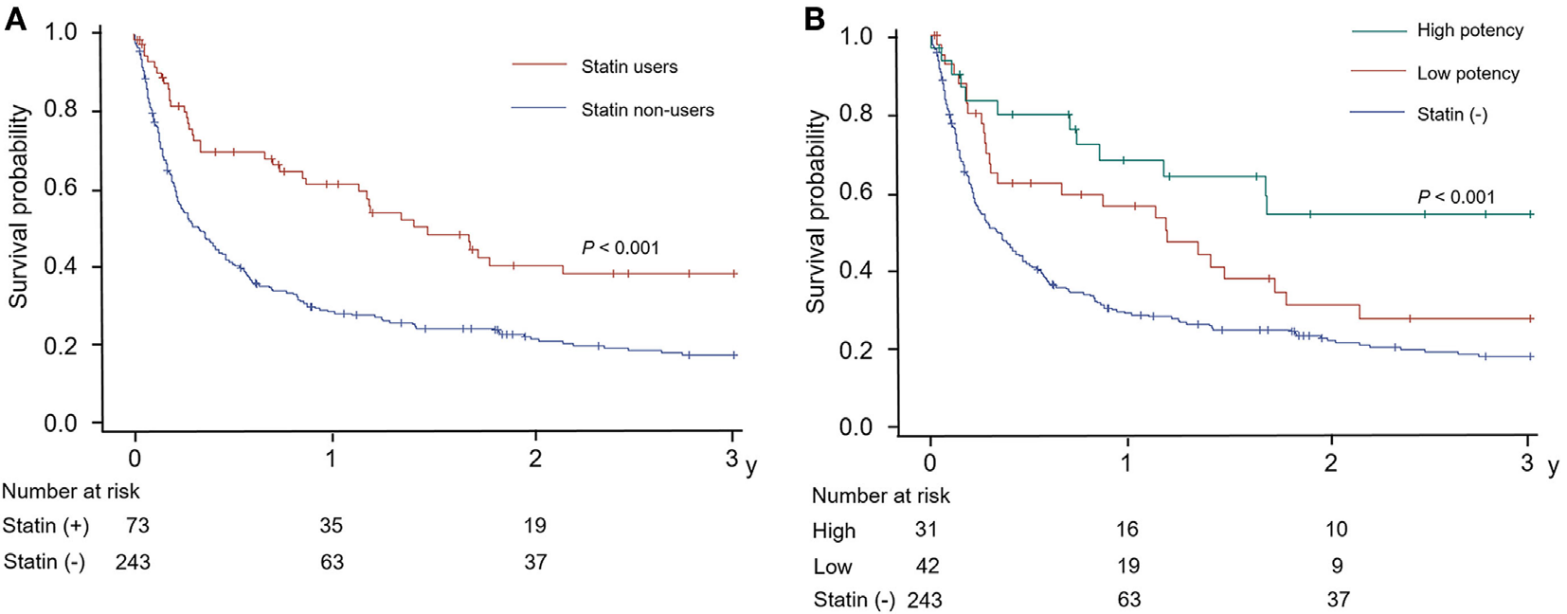
Kaplan–Meier’s curves for the survival according to **(A)** statin use and **(B)** the potency of statin used in patients with acute stroke and active cancer.

Cox-regression survival analyses revealed that statin therapy was independently associated with a lower risk of mortality (adjusted HR = 0.675, 95% CI = 0.457–0.996; Table 2; Table S2 in Supplementary Material). In addition, the higher body mass index, hemoglobin level, and platelet count were linked to reduced mortality. When considering stroke mechanisms, conventional stroke was associated with a reduced mortality, while cryptogenic stroke was associated with higher mortality. Higher NIHSS score, elevated white blood cell count, metastasis, and anticoagulation therapy were linked with increased mortality. Figure 3A is a Kaplan–Meier’s survival curve demonstrating the association between statin therapy and reduced mortality in the stroke registry cohort (Log-rank test, *p* < 0.001).

**Table 2:** Analyses of univariate and multivariate Cox’s proportional hazard model for predicting mortality.

	All subjects (*n* = 316)			
	Univariate		Multivariate^a^	
	HR (95% CI)	*p*	HR (95% CI)	*p*
Post-stroke use of statins (yes versus no)	0.491 (0.347–0.697)	<0.001	0.675 (0.457–0.996)	0.048
Potency of post-stroke statin use		<0.001		0.068
Non-users	1		–	
Low-potency statin	0.624 (0.414–0.941)	0.024	0.828 (0.523–1.309)	0.418
High-potency statin	0.336 (0.187–0.602)	<0.001	0.495 (0.269–0.910)	0.024

*CI, confidence interval; HR, hazard ratio*.

*^a^The data presented in this table were the final results of a backward stepwise multivariable Cox’s regression analysis after selecting variables that were significantly associated with the univariate analysis. The covariates used in this analysis were as follows: body mass index, white blood cell count, platelet count, modified Rankin score at 7 days, metastasis, adenocarcinoma, and post-stroke statin use. Hazard ratio for individual variables other than post-stroke statin use can be found in Table S1 in Supplementary Material*.

Additionally, as shown in Tables S1 and S2 in Supplementary Material, we further divided subjects from the stroke registry cohort by statin potency; 243 (76.9%), 42 (13.3%), and 31 (9.8%) patients were non-users, low-, and high-potency statin users, respectively. Multivariable Cox-regression survival analysis according to statin potency showed that high-potency statin was significantly associated with a lower risk of mortality (HR = 0.495, 95% CI = 0.269–0.910); low-potency statin, however, was not associated with a change in mortality (HR = 0.828, 95% CI = 0.523–1.309; Table S2 in Supplementary Material). The Kaplan–Meier curve also revealed a dose-dependent relationship between survival and statin potency, as shown in Figure 3B.

**Figure 4:**
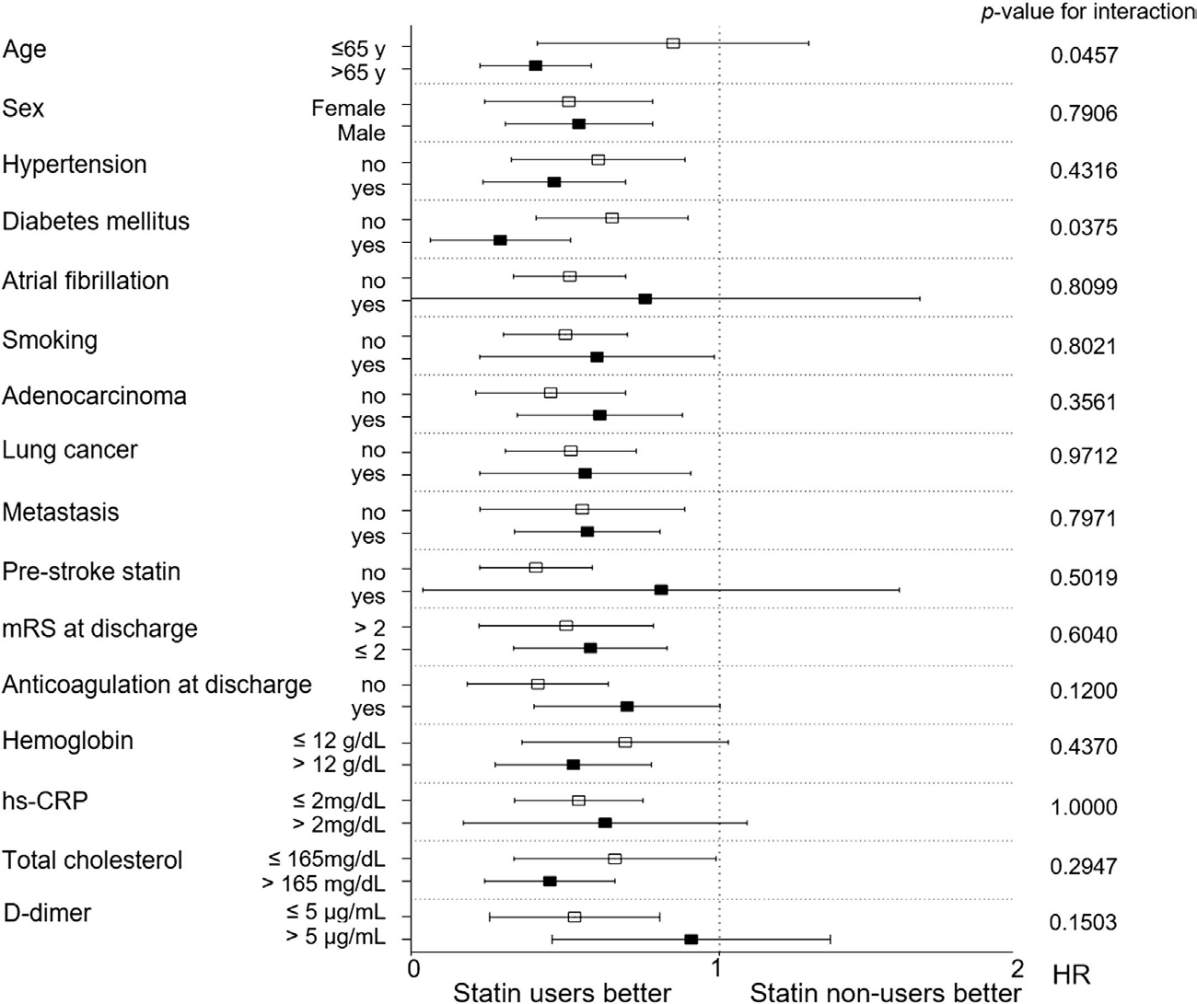
Subgroup analysis of the effect of statins on survival outcome. CRP, C-reactive protein; HR, hazard ratio; mRS, modified Rankin Scale*Subgroup analysis for D-dimer was conducted for 256 patients.

Moreover, considering the relative benefit in terms of mortality, statin therapy showed no heterogeneity according to the variables used in subgroup analyses (Figure 4). Additional analysis of 256 patients showed no significant difference in the level of D-dimer between the statin non-user (15.36 ± 16.78 µg/mL) and user groups (14.12 ± 19.41 µg/mL; *p* = 0.667). The effect of statins was evident in both the low and high D-dimer groups as seen in Figure 4.

For patients with LDL-C data (*n* = 290 patients), the LDL-C level was higher in the statin user group (109.57 ± 42.65 mg/dL) than in the non-user group (95.17 ± 34.82 mg/dL; *p* = 0.005). However, the level of LDL-C was not associated with a change in mortality (HR = 0.997, 95% CI = 0.993–1.001), while statin use significantly reduced mortality (HR = 0.477, 95% CI = 0.330–0.691).

### National Health Insurance Service Database-National Sample Cohort

A total of 36,539 subjects were diagnosed with cancer in 2007–2009 according to the NHIS nation-wide registry. Among them, 409 patients met the operational criteria for acute ischemic stroke during the follow-up period, of whom 239 (58.4%) patients were statin users (Figure 1B). The baseline characteristics did not significantly differ between statin users and statin non-users, except for a history of dyslipidemia (86.6% for statin users and 62.4% for statin non-users, *p* < 0.0001) and previous statin use (53.6% for statin users and 3.5% for statin non-users, *p* < 0.0001; Table 3). In the Cox-regression analysis, statin use was again associated with reduced mortality (adjusted HR = 0.64, 95% CI = 0.45–0.90), which persisted even after adjustment for the duration of statin use (HR = 0.86, 95% CI = 0.82–0.90 per 3 months use; mean duration 13.02 ± 19.65 months; Table 4). With respect to compliance, those who used statins for more than half of the follow-up period showed a marked reduction in mortality (adjusted HR = 0.60, 95% CI = 0.41–0.89) compared with those who did not.

**Table 3:** Baseline characteristics of the subjects from National Health Insurance System-National Sample Cohort data organized according to statin therapy use.

	Statin non-user (*n* = 170)	Statin user (*n* = 239)	*p*-Values
Age > 60 years old, *n* (%)	133 (78.2%)	176 (73.6%)	0.2866
Male sex, *n* (%)	58 (34.1%)	144 (60.3%)	0.1996
Hypertension, *n* (%)	136 (80.0%)	202 (84.5%)	0.2344
Diabetes mellitus, *n* (%)	127 (74.7%)	187 (78.2%)	0.4038
Dyslipidemia, *n* (%)	106 (62.4%)	207 (86.6%)	<0.0001
Coronary artery disease, *n* (%)	73 (42.9%)	136 (56.9%)	0.0054
Heart failure, *n* (%)	41 (24.1%)	59 (24.7%)	0.8951
Atrial fibrillation, *n* (%)	24 (14.1%)	44 (18.4%)	0.2505
Anticoagulation therapy at discharge, *n* (%)	6 (3.5%)	17 (7.1%)	0.1210
Antiplatelet therapy at discharge, *n* (%)	19 (11.2%)	51 (21.3%)	0.2505
Statin (before event),*n* (%)	6 (3.5%)	128 (53.6%)	<0.0001
Chemotherapy, n (%)	68 (40.0%)	77 (32.2%)	0.1049
Duration of statin therapy (month)—Mean (SD)	10.09 (12.57)	17.0 (20.17)	
—Median (Min–Max)	6.84 (0.03–72.17)	7.63 (0.03–70.13)	

**Table 4:** Risk of mortality after ischemic stroke in patients with cancer according to use of statin: population data from the Korean National Health Information Service-National Sample Cohort.

	Subject with ischemic stroke after cancer diagnosis				
	(*n* = 409)	Unadjusted HR (95% CI)	*p*	Adjusted HR^a^ (95% CI)	*p*
Use of statin after ischemic stroke^b^	239 (58.4%)	0.52 (0.39–0.69)	<0.001	0.64 (0.45–0.90)	0.0102
Duration of statin use^c^ (months)	13.02 ± 19.65	0.87 (0.84–0.91)	<0.001	0.86 (0.82–0.90)	<0.001
Statin adherence^d^ (%)	16.35 (0.00, 100.00)	0.53 (0.38–0.75)	<0.001	0.60 (0.41–0.89)	0.0105

*CI, confidence interval; HR, hazard ratio; Min, minimum; Max, maximum*.

*^a^Adjusted according to the following baseline characteristics: sex, age group (per 5 years), comorbidities (hypertension, diabetes mellitus, dyslipidemia, coronary artery disease, heart failure, atrial fibrillation), and medications (antiplatelet, anticoagulant)*.

*^b^Record of at least one day of statin use from onset of stroke to death or the end of the study (5 years after diagnosis of cancer). HR was calculated for the ratio per 3 months*.

^c^Total number of statin prescription days (mean ± SD) from onset of stroke to death or the end of the study. HR obtained “per 3 months.”

*^d^HR was calculated to compare between those with adherence ≥50% (*n* = 153, 37.4%) and those with adherence <50% (*n* = 256, 60.0%)*.

## Discussion

In this study, data from the multicenter stroke registry cohort showed that statin therapy was independently associated with a reduction in mortality for patients with acute stroke and active cancer. This finding is consistent with various models and external validation with the population-based NHIS-NSC database. Additionally, we included statin adherence as a variable for the analysis of the NHIS-NSC database. Good statin adherence and statin potency was associated with better survival outcomes, which agreed with the results from previous studies of statins and stroke (14, 15).

Despite the consistent evidence of a beneficial effect of statins on patients with stroke and cancer in this study, we can only speculate at the mechanism of action. Fortunately, recent studies of statins have reported various pleiotropic effects, such as anti-inflammatory effects, plaque stabilization, immunomodulation, and improvement of endothelial function (16). The neuroprotective effect attributed to anti-inflammatory effects and neurovascular modulation is now commonly accepted as an explanation for the beneficial effect of statins on acute stroke outcome for both atherosclerotic (17) and non-atherosclerotic stroke (18–20). Discovering the molecular mechanisms underlying these pleiotropic effects is currently a hot topic of research, and statins have been found to influence various cellular functions, including membrane integrity, cell signaling, and the cell cycle (21–23). The pleiotropic effect of statins in cancer was demonstrated in recent experimental studies that found statins inhibited cancer progression and promoted apoptosis by interfering with the cellular function of neoplastic cells of various cancer cell lines (24, 25). Indeed, statin therapy has been associated with better survival in various types of cancer, including advanced lung cancers (26), kidney cancer (27), prostate cancer (28), pancreas cancer (29), breast cancers (29), and ovarian cancer (30). Despite some neutral or negative reports (12, 31), large number of studies of statin use and non-cutaneous cancers have demonstrated a protective effect of statin use on either cancer mortality or incidence (32).

A potential explanation for this favorable effect of statins on cancer outcome could be related to the positive association between statin therapy and improved outcomes of thromboembolic events in cancer patients. Patients with cancer often experience thrombotic disorders, such as pulmonary thromboembolism or deep vein thrombosis (DVT) because of cancer-related hypercoagulability (33). Ischemic strokes also frequently occur in these populations and the multiple territorial infarct pattern and lack of pre-existing vascular risk factors in many of these patients suggest cancer-related hypercoagulability as the most likely mechanism for stroke in many cancer patients (34). Current practice for the treatment of such cancer-related thromboembolic disease is the use of anti-thrombogenic agents, especially anticoagulants. Although anticoagulation is an effective treatment for DVT and routinely used for the secondary prevention of ischemic strokes related to cancer-related hypercoagulability, it often fails to control ischemic stroke in many of these patients (35). Furthermore, anticoagulation therapy has a significant hemorrhagic risk due to a higher bleeding tendency in cancer patients. This creates a dilemma and need for an effective strategy in the prevention and treatment of thromboembolisms associated with cancer-related hypercoagulability. Recently, the Heart and Estrogen/Progestin Replacement Study and a randomized rosuvastatin trial found that statins reduced the occurrence of venous thromboembolism in different populations (36, 37). This finding was duplicated in studies with cancer patients, where statin use was associated with a lower prevalence of DVTs (3.5–8% in statin users versus 8–21% in non-users) (38, 39) although neutral results also exist (40). To our knowledge, the effect of statin therapy on stroke outcome in cancer patients has not been studied and this study presents novel findings on this issue.

Several limitations of this study must be noted. First, this study is based on an ethnically limited sample of Korean patients with a retrospective observational design using a prospectively collected registry and national health care sample data cohort. The information concerning potential confounders, such as health-seeking behavior, diet, and exercise, in these patients could not be attained.

Because the population compositions were different for two parts of this study, we needed to define the major conditions, “active cancer” and “acute ischemic stroke,” differently for the hospital stroke registry cohort and the national validation cohort. For the stroke registry cohort, subjects were enrolled in the registry at the time of stroke onset and followed-up for stroke recurrence and survival; however, information concerning other medical conditions was less well recorded. Thus, patients who developed cancer after stroke onset could be underrepresented in the registry. Therefore, we adopted criteria for “active cancer” from a previous study which defined having active cancer as being diagnosed with cancers or received cancer treatment within 6 months from stroke onsets or having metastatic cancer (11).

Conversely, since the NHIS database was created for reimbursement purposes and most of the information is based on diagnosis codes, additional criteria were necessary for the accurate description of clinical events, such as “acute stroke” or “active cancer.” For instance, ICD-10 stroke codes I63 (cerebral infarction) includes all ischemic strokes under one code and does not readily distinguish acute ischemic strokes from asymptomatic or chronic strokes (41). Therefore, we created operational definitions of “acute ischemic stroke” even though in this way, a selection bias could occur toward enrolling patients with more serious events and omitting those with minor events.

Since the population of this study was comprised of cancer patients, clinical information related to cancer could have provided valuable insights when interpreting our findings. However, the sample size was too small for a more detailed analysis based on the cancer location, histological type, or cancer treatment. Nevertheless, when we sub-categorized these patients into “lung cancer vs. other sites” and “adenocarcinoma vs. other histologic types,” similar results were obtained. Direct comparison of disease severities, other than metastatic status, was unachievable due to the great heterogeneity of cancer sites among individual patients. Additionally, because patients were admitted to the neurology ward for stroke treatment, information such as cancer-related performance status was not collected at the time of admission and stroke registry formation. Instead, we used metastatic status, scales for neurological functional, e.g., mRS, and laboratory findings to reflect the functional and medical condition of individual subjects. Metastatic status and mRS scores were significantly associated with increased mortality, but the beneficial effect of statins remained even after taking account of these factors in the multivariate Cox-regression analyses. For the validation cohort, due to the nature of the NHIS-NSC database, information concerning clinical scores, such as the NHISS or mRS scores, and cancer-related performance status were not available. However, chemotherapy status could be assessed. Nevertheless, the multivariate analysis still revealed a positive effect of statins on cancer survival.

It also must be noted that, because two parts of this study had some overlap in study periods, there is a chance that subjects from the stroke registry cohort were also included in the population-based validation cohort or *vice versa*. However, since the NHIS-NSC data is produced from a very large dataset, with a sampling rate of 2%, the proportion of subjects overlapping between the two cohorts is expected to be trivial.

Last, we have limited explanations for the mechanism and causal relationship between statin use and reduced mortality among the patients with stroke and cancer, due to the retrospective design of study and lack of information concerning the cause of mortality. However, our stroke registry data was obtained from multicenter stroke registries and vigorous adjustments, and subgroup analyses were performed to minimize the effect of potential selection bias. Furthermore, the nation-wide population-based cohort from the NHIS-NSC database that was used for external validation reproduced a similar result, supporting the result obtained from the stroke registry cohort.

## Conclusion

Despite continued treatment, once ischemic stroke occurs in patients with cancer, it leads to poor survival outcomes (34). The current study found that statin therapy was associated with decreased mortality in patients with active cancer who also experienced acute stroke, regardless of stroke etiology, cancer type, or location. These findings can be valuable for the treatment of acute stroke in patients with cancer, since statins could be added as a therapeutic option to improve clinical outcomes. This was a retrospective study and prospective studies including randomized trials are needed for the validation of our findings.

## Ethics Statement

The study protocol was approved and supervised by the Institutional Review Boards (IRB) of Samsung Medical Center, Korea University Guro Hospital, and Korea University Ansan Hospital. Stroke registries have been collected after obtaining informed consents from patients themselves or their family members during the admission. Since this was a retrospective observational study using anonymized database, the IRB granted a waiver to conduct this study without written informed consent.

## Author Contributions

YSK, WKS, KO, JMJ, and JL designed the study. MSP, JHL, CKK, WKS, KO, JWC, MJL, OYB, GMK, CSC, JMJ, and KHL obtained the data. JMC, JL, and WKS performed statistical analyses. YSK and JMC drafted the manuscript. JL, MSP, JHL, CKK, WKS, KO, JWC, MJL, OYB, GMK, CSC, JMJ, and KHL made critical revisions to the manuscript. WKS supervised the project.

## Conflict of Interest Statement

W-KS received study funds (not related to this study) from DAEWOONG Pharmaceutical Co., LTD., MYUNG IN PHARM. CO., LTD., Korea United PHARM. INC., and Korea University Grant (K1423491 & K1518381), J-MJ received honoraria from Pfizer Inc., and Che-il pharmaceutical company and a research fund (not related to this study) from Il-dong and DAEWOONG Pharmaceutical Co., LTD. The other authors report no disclosures.

The reviewer AB and handling editor declared their shared affiliation.
