# Supplementary material for: Effect of Statins on Survival Following Stroke in Patients With Cancer
Source: Front Neurol. 2018 Apr 24;9:205. doi: 10.3389/fneur.2018.00205 (PMC5928845; doi:10.3389/fneur.2018.00205)
Supplement: Supplementary file 1 [file data_sheet_1.docx]

**Supplementary materials:**

Effect of Statins on Survival of Stroke Patients with Cancer

Ye Sel Kim^1^, Ji-Mi Choi^2^, Juneyoung Lee ^2^, Moo-Seok Park^1^, Jun-Hwa Lee^1^, Jong-Won Chung^1^, MiJi Lee^1^, Chi Kyung Kim^3^, Jin-Man Jung^4^, Kyungmi Oh^3^, Oh Young Bang^1^, Geong-Moon Kim^1^, Chin Sang Chung^1^, Kwang Ho Lee^1^, Woo-Keun Seo^1^*

^1^Department of Neurology, Samsung Medical Center, Sungkyunkwan University, School of Medicine, Korea, ^2^Department of Biostatics, Korea University College of Medicine, Korea, ^3^Department of Neurology, Korea University Kuro Hospital, College of Medicine, Korea University, Korea, ^4^Department of Neurology, Korea University Ansan Hospital, College of Medicine, Korea University, Korea

**1. Supplementary Tables**

**Supplementary Table 1.** Baseline characteristics of the subjects according to the statin dose.

|  | **Statin-non user**  **(n = 243)** | **Low dose statin user**  **(n = 42)** | **High dose statin user**  **(n = 31)** | **Total**  **(n = 316)** | ***p* values** |
| --- | --- | --- | --- | --- | --- |
| Age | 65.41 ± 10.48 | 67.33 ± 12.53 | 70.71 ± 7.77 | 66.19±10.64 | 0.025 |
| Sex, male (%) | 144 (59.3%) | 27 (64.3%) | 46 (61.3%) | 190 (60.1%) | 0.820 |
| Body mass index, kg/m^2^ | 21.89 ± 3.72 | 23.46 ± 2.74 | 23.75 ± 3.29 | 22.28 ± 3.63 | 0.002 |
| Hypertension | 97 (39.9%) | 20 (47.6%) | 19(61.3%) | 136 (43.0%) | 0.063 |
| Diabetes mellitus | 57 (23.5%) | 11 (26.2%) | 11 (35.5%) | 79 (25.0%) | 0.340 |
| Atrial fibrillation | 25 (9.9%) | 4 (9.5%) | 3 (9.7%) | 31 (9.8%) | 0.953 |
| Smoking | 44 (29.2%) | 11 (26.2%) | 6 (19.4%) | 66 (20.9%) | 0.658 |
| Hemoglobin, g/dl | 11.42 ± 2.49 | 11.82± 1.89 | 12.62 ± 2.41 | 11.59 ± 2.43 | 0.028 |
| White blood cell count, 10^3^/µL | 9.26±7.02 | 8.51 ± 3.26 | 8.54 ± 3.26 | 9.09±6.35 | 0.685 |
| Platelet count, 10^3^/µL | 182.0 ± 105.7 | 218.0 ± 95.8 | 259.9 ± 112.5 | 194.4 ± 107.7 | 0.000 |
| C-reactive protein, mg/dl | 7.48 ± 23.78 | 3.82 ± 6.17 | 9.16 ± 20.71 | 7.22 ± 21.93 | 0.535 |
| Total cholesterol, mg/dl | 159.77 ± 45.06 | 162.57 ± 44.91 | 195.32 ± 73.08 | 163.63 ± 49.41 | 0.001 |
| Fibrinogen, mg/dl (n=288) | 365.4 ± 173.3 | 353.2 ± 137.0 | 333.7 ± 94.6 | 360.5 ± 162.3 | 0.586 |
| NIHSS at admission | 6.79 ± 6.67 | 4.79 ± 5.76 | 6.84 ± 6.68 | 6.53 ± 6.57 | 0.182 |
| Anticoagulation | 173 (71.2%) | 26 (61.9%) | 8 (25.8%) | 207 (65.5%) | <0.001 |
| Pre-stroke statin, n (%) | 4 (1.6%) | 25 (34.2%) | 25 (34.2%) | 29 (9.2%) | <0.001 |
| Stroke mechanism |  |  |  |  | <0.001 |
| Conventional | 66 (27.2%) | 23 (54.8%) | 22 (71.0%) | 111 (35.1%) |  |
| Cryptogenic | 177 (72.8%) | 19 (45.2%) | 9 (29.0%) | 205 (64.9%) |  |

Values are mean plus/minus standard deviation or number (%).; *p, comparison among all subjects including non-statin group, low-potency statin group, and high-potency statin group.; **p, comparison among stroke survivor cohort including no statin among stroke survivor, low-potency statin group, and high-potency statin group.

**Supplementary Table 2.** Analyses of univariate and multivariate Cox’s proportional hazard model for predicting mortality according to post-stroke statin use.

|  | **Univariate** | |  | **Multivariable^*^** | |  | **Multivariable^†^** | |
| --- | --- | --- | --- | --- | --- | --- | --- | --- |
|  | **HR (95% CI)** | ***p*** |  | **HR (95% CI)** | ***p*** |  |  |  |
| Age | 0.994 (0.981-1.006) | 0.313 |  |  |  |  |  |  |
| Sex, male | 0.850 (0.653-1.108) | 0.230 |  |  |  |  |  |  |
| Hypertension | 0.922 (0.708-1.200) | 0.545 |  |  |  |  |  |  |
| Body mass index, kg/m^2^ | 0.913 (0.877-0.952) | <0.001 |  | 0.908 (0.868-0.950) | <0.001 |  | 0.908 (0.868-0.950) | <0.001 |
| Diabetes mellitus | 0.818 (0.599-1.118) | 0.207 |  |  |  |  |  |  |
| Atrial fibrillation | 0.802 (0.501-1.282) | 0.356 |  |  |  |  |  |  |
| Smoking | 1.196 (0.879-1.626) | 0.254 |  |  |  |  |  |  |
| Hemoglobin, g/dl | 0.884 (0.833-0.937) | <0.001 |  |  |  |  |  |  |
| White blood cell count, 10^3^/µL | 1.025 (1.007-1.043) | 0.005 |  | 0.997 (0.995-0.998) | <0.001 |  | 1.020 (1.001-1.040) | 0.041 |
| Platelet count, 10^3^/µL | 0.997 (0.995-0.998) | <0.001 |  | 1.021 (1.002-1.040) | 0.033 |  | 0.997(0.995–0.998) | <0.001 |
| C-reactive protein, mg/dl | 1.004 (1.000-1.009) | 0.052 |  |  |  |  |  |  |
| Total cholesterol, mg/dl | 0.999 (0.996-1.002) | 0.363 |  |  |  |  |  |  |
| NIHSS at admission | 1.034 (1.014-1.055) | 0.001 |  |  |  |  |  |  |
| mRS at 7 days or discharge |  | <0.001 |  |  | <0.001 |  |  | <0.001 |
| 0 | 1 |  |  | 1 |  |  | 1 |  |
| 1 | 0.742 (0.439-1.255) | 0.266 |  | 1.029 (0.600-1.765) | 0.917 |  | 0.856 (0.502–1.460) | 0.568 |
| 2 | 1.312 (0.789-2.179) | 0.295 |  | 1.430 (0.854-2.396) | 0.174 |  | 1.371 (0.819–2.294) | 0.230 |
| 3 | 1.115 (0.673-1.847) | 0.673 |  | 1.345 (0.808-2.238) | 0.225 |  | 1.213 (0.727–2.203) | 0.460 |
| 4 | 1.766(1.068-2.920) | 0.027 |  | 1.805 (1.072-3.040) | 0.026 |  | 1.509 (0.904–2.517) | 0.115 |
| 5 | 1.814(1.135-2.899) | 0.013 |  | 2.087 (1.286-3.388) | 0.003 |  | 2.094 (1.299–3.375) | 0.002 |
| 6 | 12.189(5.655-26.273) | <0.001 |  | 88.077(34.744-223.279) | <0.001 |  | 40.876(16.619– 100.536) | <0.001 |
| Stroke mechanism |  | <0.001 |  |  |  |  |  |  |
| Conventional | 0.469 (0.350-0.630) | <0.001 |  |  |  |  |  |  |
| Cryptogenic | 2.131 (1.587-2.860) | <0.001 |  |  |  |  |  |  |
| Anticoagulation | 1.410 (1.055-1.882) | 0.020 |  |  |  |  |  |  |
| Metastasis | 3.391 (2.521-4.560) | <0.001 |  | 3.947 (2.859–5.455) | <0.001 |  | 4.033 (2.919–5.571) | <0.001 |
| Adenocarcinoma | 1.198 (0.917-1.564) | 0.185 |  | 0.714 (0.533–0.958) | 0.057 |  | 0.698 (0.520–0.935) | 0.016 |
| Pre-stroke statin use | 0.940 (0.606-1.460) | 0.783 |  |  |  |  |  |  |
| Post-stroke statin use (Yes v.s. No) No) | 0.491 (0.347-0.697) | <0.001 |  | 0.675 (0.457–0.996) | 0.048 |  |  |  |
| Post-stroke statin use (potency) |  | <0.001 |  |  |  |  |  | 0.068 |
| Non-users | - |  |  |  |  |  | - |  |
| Low-potency statin | 0.624 (0.414–0..941) | 0.024 |  |  |  |  | 0.828 (0.523–1.309) | 0.418 |
| High-potency statin | 0.336 (0.187–0.602) | <0.001 |  |  |  |  | 0.495 (0.269–0.910) | 0.024 |

^*^This model treated post-stroke statin users as a whole regardless of statin potency (yes versus no).

^†^ This model used post-stroke statin users subgrouped according to potency (non-users, low-potency statin, and high-potency statin)
